# Automated and Quantitative Assessment of Tactile Mislocalization After Stroke

**DOI:** 10.3389/fneur.2019.00593

**Published:** 2019-06-12

**Authors:** Mike D. Rinderknecht, Julio A. Dueñas, Jeremia P. Held, Olivier Lambercy, Fabio M. Conti, Leopold Zizlsperger, Andreas R. Luft, Marie-Claude Hepp-Reymond, Roger Gassert

**Affiliations:** ^1^Rehabilitation Engineering Laboratory, Department of Health Sciences and Technology, Institute of Robotics and Intelligent Systems, ETH Zurich, Zurich, Switzerland; ^2^Division of Vascular Neurology and Neurorehabilitation, Department of Neurology, University of Zurich and University Hospital Zurich, Zurich, Switzerland; ^3^Cereneo, Center for Neurology and Rehabilitation, Vitznau, Switzerland; ^4^Clinica Hildebrand Centro di Riabilitazione Brissago, Brissago, Switzerland; ^5^Institute of Neuroinformatics, University of Zurich and ETH Zurich, Zurich, Switzerland

**Keywords:** localization deficits, quantitative measurement, robotic, stroke, topesthesia, topognosis, touch, vibration

## Abstract

Topesthesia, the recognition of tactile stimulation location on the skin, can be severely affected by neurological injuries, such as stroke. Despite topesthesia being crucial for manipulating objects and interacting with the environment during activities of daily living, deficits cannot be quantitatively captured with current clinical assessments and are, as a consequence, not well-understood. The present work describes a novel automated assessment tool for tactile mislocalization in neurological patients with somatosensory deficits. We present two cases of ischemic stroke patients, describe their tactile localization deficits with the automated assessment, and compare the results to a standard manual clinical assessment. Using the automated assessment tool, it was possible to identify, locate, precisely quantify, and depict the patients' deficits in topesthesia. In comparison, the clinical assessment was not sensitive enough and some deficits would remain undetected due to ceiling effects. In addition, an MRI structural analysis of the lesion supported the existence of somatosensory deficits. This novel and quantitative assessment may not only help to raise awareness of the implications of deficits in topesthesia, but would also allow monitoring recovery throughout the rehabilitation process, informing treatment design, and objectively evaluating treatment efficacy.

## 1. Introduction

Tactile sensory function is essential for recognition and manipulation of objects (1–3). This becomes evident after neurological injuries leading to somatosensory deficits, which can affect the interaction with the environment, especially at the level of the hand (4). While there exist tools to quantify detection of tactile stimuli (5, 6), discrimination of surface properties, such as gratings (7, 8), and two-point discrimination (9), there is a lack of tools to quantify topesthesia (localization of tactile stimuli) in a standardized, sensitive, and reliable way. As a consequence, localization deficits are not routinely assessed and suffer from low awareness. According to Pumpa et al. (10), only around 6.4% of surveyed clinicians report using the Nottingham Sensory Assessment (NSA), which includes manual testing of localization rated on a trichotomous scale (i.e., absent, abnormal, normal), when testing stroke patients (11). Other 43.6% use non-standardized clinical point localization tests and more than half of the clinicians agreed that current methods for assessing somatosensory function need to be improved.

One difficulty of assessing localization, besides reproducibility of stimuli and test locations, is the visualization and quantification of localization errors. There have been few approaches resulting in hand-drawn schematics with arrows originating from the stimulus location (i.e., target) and terminating at the perceived location (12, 13), or drawings of distorted hand-shaped outlines based on responses encoded as x and y distances from the target (14). Using these approaches, it was possible to identify in stroke patients both shifted and compressed somatotopic representations preserving a relative topography (14) as well as severely distorted somatotopic representations without systematic patterns (12, 13). Nevertheless, these approaches suffer from limitations similar to the ones of clinical assessments. As they are still administered manually, stimulation locations are not well-defined, and recording of perceived locations may be cumbersome.

Recently, we presented an novel automated approach to quantify and visualize tactile mislocalization on the hand (15). This system was designed to address some of the aforementioned limitations and consists of two wearable gloves which can provide well-controlled stimuli at reproducible locations and a touchscreen to directly indicate with the non-tested hand the precise location of perception on the tested hand. The present work aimed at testing the assessment as a proof of concept in two chronic stroke patients and illustrating the capability of the assessment to detect and describe deficits. The results from the automated assessment are compared with a clinical assessment for localization, based on the subscale of the Nottingham Sensory Assessment (NSA) (11). We expect that the automated assessment will reveal localization deficits beyond of what the clinical assessment can detect, and that these results can be supported by a magnetic resonance imaging (MRI) structural analysis showing lesioned brain areas critical for somatosensory processing.

## 2. Case Reports

The ReHaptic Glove system was designed as a novel automated assessment tool for tactile mislocalization in neurological patients with somatosensory deficits. We studied two chronic stroke patients that suffer from impaired tactile localization using the automated assessment tool. The magnetic resonance imaging (MRI) of the head analyzed here was performed as part of the standard stroke protocol of the Department of Neurology at the University Hospital Zurich. The scans included T1, T2, and fluid attenuated inversion recovery imaging, all with standard parameters on a 3.0 T Siemens (Erlangen, Germany) scanner.

### 2.1. Case 1

**Figure 1:**
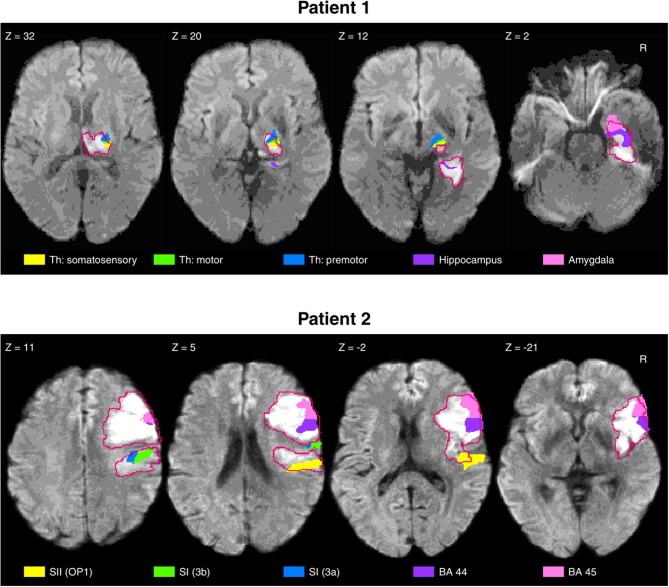
Structural MRI lesion analysis for patients 1 and 2. The lesion of patient 1 **(Top)** covered parts of the hippocampus and amygdala, but most importantly, regions of the thalamus with projections to sensorimotor regions. The lesion of patient 2 **(Bottom)** covered great extent of the Brodmann areas 44 and 45, and parts of the primary (SI) and secondary somatosensory cortices (SII), thus strongly affecting the sense of touch. The lesion borders (red outlines) were drawn on the original 3D images, using MRIcron software (21).

Patient 1, a 67-years-old (at day of hospitalization) right handed man, was hospitalized in 05/2013 with multiple cerebral infarctions in the right posterior cerebral artery territory. Stroke MRI showed ischemic lesions in the right thalamus, parts of the internal capsule, and the medial occipitotemporal gyrus (Figure 1). In addition, small subacute focal occipital and cerebellar lesions were observed on the left side. The initial neurological examination showed minimal motor deficits and severe sensory loss in the upper left extremity, with a National Institutes of Health Stroke Scale [NIHSS; Brott et al. (16)] of 4/4 points for the left arm and 2/2 points for severe sensory loss. Following 6 weeks of inpatient neurorehabilitation, the patient was discharged with small improvements of his motor and sensorimotor deficits. On enrollment in this study, 47 months after the stroke, the patient is able to perform fine motor tasks with the left (contralesional) hand, scoring 9/14 points on the hand section of the Fugl-Meyer Assessment of the Upper Extremity [FMA-UE; Fugl-Meyer et al. (17)]. The patient is able to use the left (contralesional) hand in daily life to grasp and manipulate objects. However, when the patient does not direct attention to the grasp, this may result in an unintentional release of the object or increased grip force. The patient reported subjective discomfort with tactile localization for the left (contralesional) hand. No other neurological deficits were observed by the neurological examination on study enrollment.

### 2.2. Case 2

Patient 2, a 68-years-old (at day of hospitalization) right handed woman, was hospitalized in 12/2016 following an acute right middle cerebral artery infarction. Ischemic lesions were detected in the right inferior frontal gyrus (pars opercularis), insula, lateral parts of the pre- and post-central gyrus, superior temporal gyrus, supramaginal gyrus, and corona radiata (Figure 1). On admission, the patient suffered from severe motor deficits (NIHSS left arm: 4/4 points) and severe sensory loss (NIHSS sensory: 2/2 points) of the upper extremity. Twelve weeks of inpatient neurorehabilitation resulted in significant improvements of motor function, while the somatosensory deficits persisted. When the patient is enrolled in this study 12 months post-stroke, fine hand movements are possible according to the FMA-UE (hand section: 12/14 points). The patient is able to use the left (contralesional) hand in daily life and has sufficient motor strength to hold and manipulate objects. When out of his visual field, the hand would spontaneously release a grasped object without the patient's awareness. The patient reported perceiving severe localization deficits for the left (contralesional) hand. On study enrollment, no other neurological deficits were observed by the neurological examination.

### 2.3. Investigations

Prior to the assessments, both patients received information about the investigations. Written informed consent was obtained from the patients for performing the investigations and the publication of this case report. This case study was conducted in accordance with the Declaration of Helsinki and was approved by the Cantonal Ethics Commission of the Canton of Zurich (Req-2016-00285). The investigations of patient 1 and patient 2 were performed approximately 47 and 12 months after the insult, respectively.

**Figure 2:**
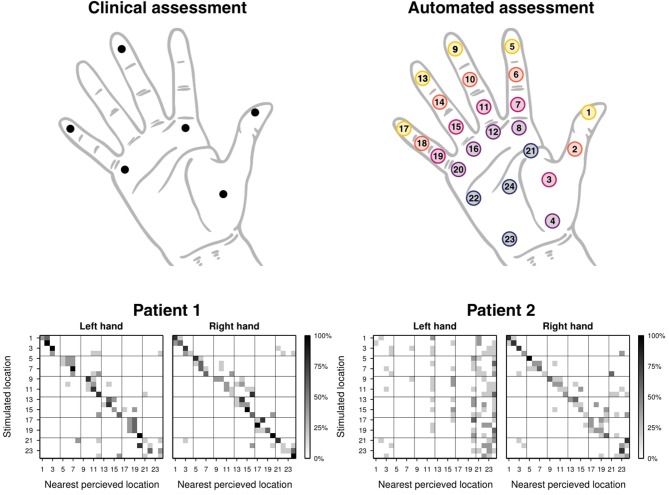
Test locations of the clinical and automated assessments, and localization confusion matrices of patients 1 and 2 (both with right hemispheric stroke) resulting from the automated assessment. The test locations of the manual clinical assessment are shown in the top left schematic hand, and the ones for of the automated assessment in the top right schematic hand (the colors yellow to blue indicate distal to proximal). The localization confusion matrices **(Bottom)** show the percentage of discretized reported location of perception based on the nearest stimulation location. Note that for correct interpretation of the matrix, the numbering of the test locations should be related to the specific locations on the hand **(Top Right)**, instead of interpreting the distance of the gray pixels to the diagonal of the matrix, as neighboring numbers do not necessarily correspond to neighboring locations.

The localization deficits were first manually assessed, based on the instructions of the localization subscale of the Nottingham Sensory Assessment (NSA) (11) and the locations indicated in Stolk-Hornsveld et al. (18). The tests were performed using a neuropen (19) for patient 1, and a straightened paper clip for patient 2. The following locations were tested: volar sides of distal phalanges of the 1st, 3rd, and 5th digit, distal volar side of the 2nd and 5th metacarpal, and center of the thenar eminence (Figure 2, top left). The patient was asked to indicate the location of the tactile stimulation on the tested hand with closed eyes. A localization error of 2 cm was allowed. Each location was tested three times and rated on a scale from 0–2 (0: absent, incorrect on all three repetitions; 1: impaired, correct for some of the three repetitions; 2: normal, correct for all three repetitions). Both hands were tested in random order.

The automated assessment was conducted with the ReHaptic Glove, a research prototype system based on two gloves with haptic feedback and a touchscreen computer (15). Each glove can apply well-controlled, reproducible vibrotactile stimuli on 24 locations distributed on the volar side of the hand (Figure 2, top right). The task consisted in localizing vibrotactile stimuli by indicating the perceived location directly on a schematic hand displayed on the touchscreen. No visual cues were given on the location of the stimulation. Stimuli lasted 1.5 s at a vibration frequency of around 150 Hz, thus activating rapidly adapting units (i.e., mainly Pacinian corpuscules) (20). The pressure of the vibrotactile stimuli provided by the eccentric rotating mass vibrators was not individually controlled. However, through the design of the device and its elastic properties, a snug fit can be achieved for a certain range of hand sizes (hand length of around 170–210 mm), allowing the vibrators to maintain contact with the skin while vibrating with an amplitude of around 1 mm. Each location was tested five times in pseudo-random order, leading to a total of 120 trials. The patients received up to three attempts (i.e., repetitions of the trial) for each trial when they either did not perceive the stimulus or missed it due to inattention. Both hands were tested in random order. The procedure of the assessment (apart from assisting the patient in putting on the glove and starting the software) is fully automatized and the graphical user interface of the software directly prompts the patient to provide his/her response after stimulation. No further intervention and no technical skills are required by the experimenter. Based on the coordinates recorded from the indicated locations by the patient, three outcome measures can be calculated: distance, offset, and spread (15). Both the distance and the offset can be interpreted as a measure of accuracy. The distance represents the average of the geodesic errors (locally length-minimizing curve within the schematic hand) of coordinates reported for a specific location with respect to the stimulation location, and the offset represents the geodesic error of the center of the coordinates reported for a specific location with respect to the stimulation location. In contrast, the spread can be interpreted as a measure of precision, as it represents the average of the geodesic distance between the reported coordinates and their center. The offset and the spread were used to create distortion maps representing the distorted localization perception on the hand of a subject. Based on discretization of the reported perceived location to the nearest stimulation location a confusion matrix was calculated (Figure 2, bottom). The diagonal of the matrix is referred to as correct localization rate. Technical details of the assessment tool and the outcome measures as well as the results of a pilot study with young, neurologically intact subjects were described by Rinderknecht et al. (15).

Additionally, we conducted an MRI-based structural analysis of the lesions for both patients. The analysis of the lesions was based on T2-weighted images with the greatest extent of damage noticeable. The lesion borders were drawn in native space, using MRIcron software (21). The anatomical scans and lesion volumes were then normalized to a standard brain template using the SPM12 toolbox (22) of the Wellcome Trust Centre for Neuroimaging (University of London, London, United Kingdom) running in MATLAB 2016b (The MathWorks, Inc., Natick, MA, USA). The normalized lesion regions were used as region of interest (ROI) to identify the affected regions using the Anatomy toolbox (23). The regions affected were identified by overlapping the ROIs with the probabilistic cytoarchitectonic maps (24). For the purpose of this study, we focused our analysis on the regions critical for tactile sensation (thalamus, primary and secondary somatosensory cortex (parietal operculum), and posterior parietal cortex).

### 2.4. Results

**Figure 3:**
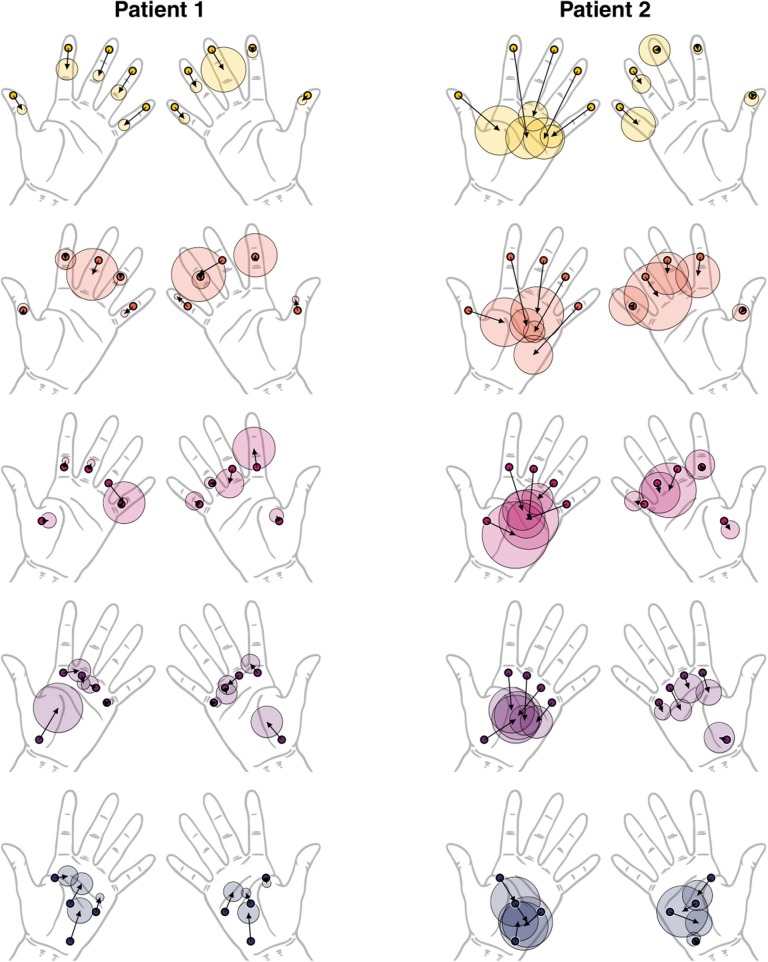
Visualization of localization performance of patients 1 and 2 (both with right hemispheric stroke). The arrows originate from the stimulus location (thick black circles) and end at the center of the perceived locations. The length of the arrow corresponds to the offset metric (distortion of perception). The semitransparent circle around the arrow endpoint corresponds to the spread of the responses (uncertainty of perception). The colors yellow to blue indicate distal to proximal distribution of locations and is in accordance to Figure 2 (top right).

Clinical assessment scores are listed in the first part of Table 1. For the automated assessment, the outcome measures offset and spread were indicated for each location in the second part of Table 1, together with all outcome measures averaged across all 24 tested locations for a rough comparison. Detailed outcome measures for the correct localization rate follow in Figure 2 (bottom), and Figure 3 shows a graphical illustration of the individual offset and spread metrics.

**Table 1:** Results of the clinical and automated localization assessments for patients 1 and 2 (both with right hemispheric stroke).

**Clinical assessment**		**Patient 1**		**Patient 2**	
		**Left**	**Right**	**Left**	**Right**
**Correct localization [0–2]**		**(contralesional)**	**(ipsilesional)**	**(contralesional)**	**(ipsilesional)**
Digit I, distal phalanx		2	2	1	2
Digit II, metacarpal		2	2	0	2
Digit III, distal phalanx		2	2	0	1
Digit V, distal phalanx		2	2	1	2
Digit V, metacarpal		2	2	1	1
Thenar eminence		2	2	0	2
Total score		12	12	3	10
**Automated assessment**		**Patient 1**		**Patient 2**	
		**Left**	**Right**	**Left**	**Right**
Offset ± spread [cm]^*^		**(contralesional)**	**(ipsilesional)**	**(contralesional)**	**(ipsilesional)**
Digit I	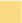 Location 1	1.8 ± 0.5	0.8 ± 0.5	6.5 ± 2.5	0.4 ± 0.8
	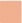 Location 2	0.2 ± 0.6	1.2 ± 0.4	4.2 ± 2.5	0.3 ± 0.9
	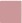 Location 3	0.7 ± 0.8	0.7 ± 0.7	3.5 ± 3.4	1.2 ± 0.9
	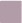 Location 4	4.1 ± 2.5	2.5 ± 1.6	4.1 ± 2.5	0.9 ± 1.6
Digit II	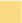 Location 5	2.3 ± 1.1	0.4 ± 0.6	9.8 ± 2.2	0.2 ± 0.5
	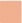 Location 6	0.3 ± 1.1	0.2 ± 2.2	7.8 ± 1.7	2.1 ± 2.3
	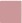 Location 7	0.7 ± 0.4	2.0 ± 2.2	5.0 ± 2.0	0.3 ± 1.5
	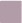 Location 8	1.8 ± 1.2	1.2 ± 1.0	4.0 ± 2.3	2.3 ± 1.3
Digit III	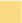 Location 9	3.1 ± 0.6	2.6 ± 2.3	7.8 ± 1.5	0.2 ± 1.6
	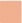 Location 10	1.7 ± 2.7	8.2 ± 2.8	5.7 ± 2.7	1.3 ± 2.2
	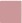 Location 11	0.7 ± 0.4	1.6 ± 1.5	5.4 ± 3.1	2.6 ± 2.8
	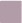 Location 12	0.9 ± 0.7	1.6 ± 1.0	4.7 ± 1.5	1.5 ± 1.5
Digit IV	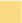 Location 13	2.8 ± 0.8	2.0 ± 0.6	8.4 ± 2.1	1.7 ± 1.0
	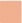 Location 14	0.2 ± 0.7	0.3 ± 0.9	7.0 ± 1.1	2.6 ± 3.5
	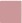 Location 15	3.6 ± 2.1	0.2 ± 0.6	2.3 ± 1.6	1.1 ± 2.1
	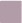 Location 16	0.8 ± 0.9	0.7 ± 1.1	3.8 ± 2.5	2.7 ± 1.1
Digit V	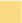 Location 17	3.0 ± 0.6	1.6 ± 0.6	5.3 ± 1.2	2.6 ± 1.8
	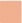 Location 18	1.2 ± 0.4	1.5 ± 0.3	6.9 ± 2.0	0.4 ± 2.0
	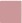 Location 19	0.4 ± 0.4	0.6 ± 0.9	4.8 ± 2.0	1.2 ± 1.0
	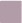 Location 20	0.4 ± 0.3	0.3 ± 0.5	2.7 ± 1.7	1.1 ± 0.8
Palm	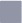 Location 21	1.4 ± 1.0	0.6 ± 0.4	3.1 ± 2.5	2.4 ± 1.5
	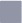 Location 22	1.7 ± 0.4	2.4 ± 1.0	2.3 ± 2.5	3.4 ± 1.4
	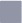 Location 23	3.6 ± 1.3	3.0 ± 1.1	2.2 ± 2.0	0.3 ± 0.7
	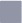 Location 24	2.5 ± 1.1	1.3 ± 0.5	2.4 ± 2.6	1.7 ± 2.6
Mean offset [cm]^*^		1.7 ± 1.2	1.6 ± 1.6	5.0 ± 2.1	1.4 ± 1.0
Mean spread [cm]^*^		0.9 ± 0.7	1.1 ± 0.7	2.2 ± 0.6	1.6 ± 0.8
Mean distance [cm]^*^		1.9 ± 1.3	1.8 ± 1.3	5.5 ± 2.0	2.1 ± 1.0
Mean correct localization rate [%]		42.5 ± 37.0	46.7 ± 33.2	6.7 ± 14.0	43.3 ± 27.5

*Clinical assessment: performance is rated as absent (0), impaired (1), or normal (2). Automated assessments: Reported are offset ± spread for each tested location and hand, and mean ± standard deviation for the different outcome measures, across all tested locations. The correct localization rate is based on discretization of perceived location to the nearest stimulation location*.

* *Normalized for a hand length of 17 cm, measured from the tip of the middle finger to the wrist crease*.

Patient 1 obtained maximum scores for both hands and all locations in the clinical assessment (upper half of Table 1. The overall localization performance, as assessed by the automated assessment, was similar for both hands, with a tendency of worse performance for the left (contralesional) hand. The inspection of the performance at individual locations provides further insights on the localization deficits. For this, the distortion maps illustrating perceived locations using the outcome measures offset and spread (Figure 3, left) and the confusion matrices (Figure 2, bottom left) were computed as described by Rinderknecht et al. (15). Figure 3 (left) shows a systematic shift from distal phalanges to intermediate phalanges in all five fingers of the left (contralesional) hand in patient 1. In the right (ipsilesional) hand the shift was less systematic and less pronounced. Furthermore, it becomes visible that some locations are systematically perceived on a wrong finger (e.g., intermediate phalanx of the middle finger). The stimulus was erroneously perceived in two out of five trials on the intermediate phalanx of the ring finger, as shown in the confusion matrix (Figure 2, bottom left). The grid lines of in the confusion matrix delimit the fingers [digit I (locations 1–4): distal and proximal phalanges and distal and proximal aspects of metacarpal; digits II–V (locations 5–20): distal, intermediate, proximal phalanges, and distal aspect of metacarpals] and facilitate the inspection of stimuli located on wrong fingers on the confusion matrix. According to the MRI structural analysis, a substantial lesion was found in the right thalamus, covering regions with projections to somatosensory, motor and pre-motor cortices, with lesion percentages of 62.5, 55.8, and 48.2%, respectively. In addition, the lesion of patient 1 covered the hippocampus and amygadala, both on the right hemisphere, with a lesion percentage of 74 and 55% of each region. The duration of the automated assessment for patient 1 was 15.1 and 11.5 min for the right and left hand, respectively.

Patient 2 showed severe localization deficits for the left (contralesional) hand and partial deficits for the right (ipsilesional) hand based on the clinical assessment. Our automated assessment showed severe localization deficits for the left (contralesional) hand. The outcome measures for the right (ipsilesional) hand of patient 2 provided by the automated assessment were in a similar range as the outcome measures of patient 1. The scores of the clinical assessment as well as the automated assessment are summarized in Table 1. Patient 2 showed severe deficits in localization in all fingers from distal toward the center of the palm in the left (contralesional) hand, as visible in the distortion map (Figure 3, right). Furthermore, the patient had difficulties localizing the vibrotactile stimuli with certainty as shown by the large spread, despite being able to clearly detect their presence. These deficits are reflected in the confusion matrix for the left hand (Figure 2, bottom right), where most perceived locations are in the rightmost squares, representing the palm instead of having a more diagonal appearance. The right (ipsilesional) hand of patient 2 showed only a minor distoproximal shift from the intermediate phalanges and from the distal aspect of the metacarpals. Patient 2 was unable to localize the stimulus (or did not perceive it) after three attempts in one out of five trials for locations 5 and 24 on the left hand and locations 3, 14, 18, and 23 on the right hand. According to the MRI structural analysis, the lesion covered important somatosensory regions, such as the finger areas of regions 3a and 3b of the primary somatosensory cortex (SI), and of the secondary somatosensory cortex (SII) in the parietal operculum. The lesion covered 25.9%, 16.3%, and 61.5% of the regions 3a, 3b, and operculum 1 (OP1), respectively. Moreover, patient 2 presented a prominent lesion in the right Brodmann areas 44 and 45, with a lesion percentage of 88.5% and 80.7% of each area. For patient 2 the duration of the automated assessment was 15.5 min (right hand) and 21.2 min (left hand).

## 3. Discussion

The aim of this case study was to test an automated tool to assess mislocalization in stroke patients and to illustrate its utility and potential as a sensitive assessment tool for clinical applications. The two case reports illustrate that using the present instrumented approach allowed detecting, quantifying, and visualizing deficits in topesthesia, beyond of what is possible with the clinical assessment for localization. Furthermore, the presence of somatosensory deficits can be explained with the MRI-based lesion location analysis.

### 3.1. Case 1

In the case of patient 1, the clinical assessment could not detect any somatosensory deficits, and the subjective discomfort with tactile localization could not be captured. In addition, it was observed that the patient had problems when holding objects when not paying attention, which is a strong indication for lasting somatosensory deficits (25). In contrast to the clinical assessment, Figure 3 (left) shows a systematic shift of perceived locations at the fingertips of left (contralesional) hand from distal to proximal, when assessed with the automated assessment. As a matter of fact, the correct localization rate is as low as 12.0% for fingertips (locations 1, 5, 9, 13, and 17) of the left hand, which explains the large standard deviation in Table 1. This is far below the correct localization rate of 70.0±32.7% for the left hand in healthy adults (15). Compared to the left (contralesional) hand, the right (ipsilesional) hand performed on average better in terms of distance, offset, and correct localization rate, yet, still worse compared to healthy subjects [e.g., 48.0% for the fingertips compared to 70.7±32.3% for healthy subjects shown by Rinderknecht et al. (15)]. The deficit in topesthesia is probably a consequence of the lesion in the ventral posterior nucleus of the thalamus (Vc or VPL) which is the thalamic principal somatic sensory nucleus (26), which forwards the tactile and vibration information from the medial lemniscus to the somatosensory cortex (25). The exact projections of the Vc has not yet been described but a first description of thalamocortical connectivity using diffusion tensor imaging identifies its connection the somatosensory cortex (27). There does not exist a detailed somatotopy in this nucleus so far. Yet, the similar deficits on the distal phalanges of neighboring fingers revealed by our assessment could suggest some somatotopic organization of the finger representation in the lesioned thalamic region. The group of Lenz has investigated the tactile properties of Vc neurons in humans prior surgical procedures for motor disorders and shown a clear organization of face, arm, hand, and fingers [Chien et al. (28), for review]. Microstimulation in the recording sites evoked tactile sensations localized in the same body parts.

### 3.2. Case 2

For patient 2 the clinical localization assessment revealed severe deficits in the left (contralesional) hand and minor deficits in the right (ipsilesional hand). While the clinical assessment resulted in a very low score for the left (contralesional) hand showing only the ratio of correctly localized stimuli and providing very limited information on the type of distortion, the automated assessment uncovered the detailed morphology and severity of the somatotopic distortions. The perception of tactile stimuli on the left hand was completely reduced to the palmar area, as visible in Figure 3 (right). Tactile stimulation on all five fingers were consistently perceived more proximal on the palm. The fact that all the points tested on the fingers were perceived on the palm would strongly suggest that the fingers are not represented anymore. However, Figure 3 (right) shows that the perceived locations for the fingers on the palm still follow the neighboring order of the fingers to a certain extent, based on which it could be argued that the representation is severely shrunken but not entirely absent or non-systematically distorted. This severe deficit in topesthesia clearly results from damage in SI, specifically in area 3b which is relevant to the sensation of tactile stimuli (human touch and vibrotactile stimuli, respectively) on the fingers (29, 30). Partial damage in SII could explain some minor systematic bilateral deficits (e.g., distoproximal shift of stimulus locations 6, 10, 14 and 8, 12, 16, 20 on the ipsilesional hand) (31–35). These deficits could also be due to reduced attention, as it has been shown that there is enhanced activation in SII when directing attention to tactile stimuli (36). Thus, SII being involved in tactile memory processes (i.e., tactile working memory) would explain its importance for the present task, as a vibrotactile stimulus provided by the automated system lasted only 1.5 s and the patients usually tended to respond after the stimuli. Similar to patient 1, the case presented by Birznieks et al. (12) which had also a large stroke (right middle cerebral artery infarction mainly affecting parietal and temporal lobe) showed severe mislocalization due to affected SI.

### 3.3. Limitations

One minor limitation of the comparison between the clinical assessment and the automated assessment arises from the stimulation type. While in the clinical assessment the tactile stimulation consists in touching or poking the patient's hand with a thin tool (activating more slowly adapting mechanoreceptors), the stimulation of the automated assessment consists in tactile vibration. This tactile vibration is mainly perceived superficially and is applied on locations with more subcutaneous tissue (fat) unlike the vibration stimuli applied with a tuning fork placed upon a bony prominence. Nevertheless, both touch and vibrotactile sensation are conveyed via the dorsal column–medial lemniscus pathway. Furthermore, the task is to locate suprathreshold stimuli on the skin requiring cortical analysis to provide more complex interpretation of primary somatosensory information instead of just assessing the absence of touch appreciation or the precise nature of the stimuli. Thus, whether suprathreshold tactile stimulation is in the form of touch or tactile vibration should not be crucial for the interpretation of the assessment outcomes.

The voxel-based structural lesion analysis based on MRI scans did not allow to identify individual finger areas in the brain within the ROIs for these two case reports. This is due to two limitations: low spatial resolution of the 3.0 T scanner and the fact that the Anatomy toolbox does not yet include templates with individual finger areas. More studies using scanners with higher spacial resolution [e.g., (29, 30, 37)] using a 7.0 T scanner to map finger somatotopy in SI) and well-controlled stimulation will provide more detailed insights in somatotopy and allow to improve these type of analyses (38).

## 4. Conclusion

In order to better understand the consequences of post-stroke distorted somatotopic representations leading to deficits in tactile localization and its importance to functional recovery, it is necessary to be able to quantitatively assess and monitor these deficits. While current clinical assessments can detect the existence of major deficits in topesthesia, they still fail to sensitively quantify them. The two case reports on stroke patients presented in this work demonstrate the utility and advantages of using a new, more sophisticated assessment tool to quantify mislocalization. This automated, technology-assisted assessment allows to assess topesthesia on the volar side of the hand in a standardized way with well-controlled stimulation for higher repeatability and provides continuous outcome measures on ratio scales. The computed visual representations allow clinicians to get a very detailed overview of the distorted perception for the entire hand at a glance. Thus, the automated assessment is expected to accelerate clinical routine examinations, while providing richer information and facilitating the interpretation compared to current clinical scales. In the two case reports, the proposed automated assessment was able to uncover deficits where clinical assessments could not due to ceiling effects and low sensitivity, and further suggested the presence of deficits in the ipsilesional hand, which is often referred to as the “non-affected” side in stroke patients. Furthermore, the existence of the revealed deficits in both patients could be related to the lesion location based on a voxel-based structural MRI analysis. These two cases show that strokes affecting the dorsal column—medial lemniscus pathway at different levels (e.g., ventral posterolateral nucleus of the thalamus, internal capsule, or SI) can result in vibrotactile localization deficits. In cross-sectional studies such an assessment tool combined with MRI could allow a more detailed analysis to improve knowledge about somatotopy in different brain areas, as for example systematic distortions on neighboring fingers could be an indicator for co-located finger representations in the brain regions.

Different studies have shown that mislocalization can persist many years after stroke (12, 14, 39) and only little research has investigated recovery. However, there exists recent evidence for potential normalization of distorted somatotopic representations in chronic stroke patients (40). Hence, therapies for deficits in topesthesia should be explored and whether recovery can be accelerated through targeted tactile stimulation. The present automated approach to assess tactile mislocalization provides a sensitive tool, which could inform the selection of the most appropriate therapy for the individual patient or the design of new customized and targeted rehabilitation approaches for topesthesia. The use of this tool will allow conducting systematic longitudinal studies to monitor detailed changes in localization deficits, which, combined with normative data and lesion analyses using MRI, may shed light on which patients are responders to specific therapies, on whether there exists a critical time window for interventions, and on the evolution of recovery in patients with somatosensory deficits. Furthermore, the presented tool combined with adaptive algorithms could be directly used for closed-loop patient-tailored somatosensory rehabilitation, as the system allows to assess tactile localization performance and the interface allows to provide immediate feedback to the patient.

## Data Availability

All datasets generated for this study are included in the manuscript and/or the supplementary files.

## Ethics Statement

Prior to the assessments, both patients received information about the investigations and gave their written consent. This case study was conducted in accordance with the Declaration of Helsinki and was approved by the Cantonal Ethics Commission of the Canton of Zurich (Req-2016-00285).

## Author Contributions

MR, JH, OL, and RG designed the study. MR developed the methodology and conducted the instrumented assessment, performed the analysis, and drafted the manuscript. JD performed the MRI voxel-based lesion analysis. MR, JD, M-CH-R, and FC interpreted the results. JH recruited the patients and conducted the clinical assessment. LZ documented the medical histories of the two patients. All authors revised the manuscript and approved the final version.

### Conflict of Interest Statement

AL is scientific advisor for Hocoma AG (Volketswil, Switzerland), which develops rehabilitation technology. The ReHaptic Glove research prototype was developed at the Rehabilitation Engineering Laboratory at ETH Zurich and is not commercially available. The remaining authors declare that the research was conducted in the absence of any commercial or financial relationships that could be construed as a potential conflict of interest.
